# Establishment and Development of Oral Microflora in 12–24 Month-Old Toddlers Monitored by High-Throughput Sequencing

**DOI:** 10.3389/fcimb.2018.00422

**Published:** 2018-12-04

**Authors:** Fei Li, Danying Tao, Xiping Feng, May. Chun. Mei Wong, Haixia Lu

**Affiliations:** ^1^Department of Preventive Dentistry, Shanghai Ninth People's Hospital, College of Stomatology, Shanghai Jiao Tong University School of Medicine, Shanghai, China; ^2^National Clinical Research Center for Oral Diseases, Shanghai Key Laboratory of Stomatology & Shanghai Research Institute of Stomatology, Shanghai, China; ^3^Dental Public Health, Faculty of Dentistry, University of Hong Kong, Hong Kong, China

**Keywords:** toddlers, early childhood caries, oral microbial diversity, high-throughput sequencing, *Streptococcus*

## Abstract

A cohort study was conducted to evaluate oral microbial diversity among toddlers aged 12–24 months, and to describe the dynamic processes of colonization, development, and stabilization of the oral microflora during tooth eruption using high-throughput sequencing technology. A total of 20 healthy toddlers aged 12 months were included at baseline and followed up through 18–24 months. Clinical oral examinations of dental caries status and visible plaque index were carried out at three follow-up time points. Pooled supragingival plaque biofilm samples were also collected at ages 12, 18, and 24 months. Plaque biofilm DNA was extracted and analyzed by MiSeq sequencing. A total of 18 toddlers completed three follow-ups. At 12 months of age, all the toddlers only had eruption of the anterior teeth, without dental caries. At ages 18 and 24 months, one and two toddlers showed two and three teeth with carious white spots, respectively. Depth, Good's coverage, and sample size of sequencing were reasonable. The dominant bacterial genera in the oral cavity of 12-month-old toddlers were *Capnocytophaga, Neisseria, Streptococcus, Kingella*, and *Leptotrichia*; the oral microflora composition was relatively stable by 18 months of age and included *unclassified Enterobacteriaceae, Selenomonas, Prevotella, Leptotrichia*, and *Veillonella* as the dominant genera; *unclassified Enterobacteriaceae, Streptococcus, Neisseria, Leptotrichia*, and *Selenomonas* were the dominant genera by 24 months. There were significant differences among microbial compositions in the oral cavities of 12, 18, and 24-month-old toddlers, with relatively small differences observed between the 18 and 24 months samples. In conclusion, oral microbial community of toddlers showed a trend of dynamic development. Significant differences in oral microbial diversity among toddlers aged 12–24 months were observed, while the microbial diversity differences among toddlers aged 18–24 months tended to be more similar. The findings indicated that the oral microbial community gradually matures and tends to stabilize with the growth and development of toddlers.

## Introduction

The oral ecosystem comprises a diverse group of bacteria that have been selected over the course of evolutionary co-adaptation between oral bacteria and the host. These include indigenous oral microflora, supplemental oral microflora, and transient oral microflora. The establishment of normal oral microflora and their succession in children is closely related to age, eating habits, and oral hygiene habits as well as the indigenous oral microflora of their parents. Newborns are toothless and develop deciduous teeth as they grow. This process includes the establishment of deciduous dentition, a process during which microflora in the oral cavity also undergo changes. Investigating the characteristics of bacterial colonization during the process of tooth eruption in toddlers will help to elucidate the conditions under which microecological balance in the oral cavity may be maintained. Understanding these events will contribute to the prevention of dysbiosis-induced diseases such as early childhood caries (ECC).

Studies on early colonization by oral bacteria date back to 1965, when McCarthy performed dynamic observations of the indigenous bacterial flora in newborns and toddlers (McCarthy et al., 1965). Since the 1970s, related research has focused on the isolation, culture, and identification of *Streptococcus* species in the oral cavity of toddlers (Carlsson et al., 1970; Whiley et al., 1998). In the 1990s, improvements in bacterial culture technology, particularly anaerobic culture technology, and the application of molecular methods to bacterial studies, made possible the gradual emergence of research on early colonization of anaerobic bacteria in toddlers (Könönen et al., 1999). However, most of these studies only focused on the dominant bacteria in the oral cavity and the colonization characteristics of one or several bacteria. Since 2000, an increasing number of researchers have supported the “ecological plaque hypothesis”(Marsh, 1994)—that the microbiology of the oral microenvironment should be viewed as a whole when interpreting biological behavior. In light of this perspective, microbiome research has become a research trend, in contrast to conventional research methods focusing on single bacterial species. An overall understanding of the diversity, distribution, and succession of oral microflora in toddlers is more meaningful from an ecological standpoint.

To date, most of the studies on ECC are cross-sectional, with very few cohort studies; moreover, they often begin after the occurrence of ECC, and do not include a description of oral microbial diversity in the early stages following tooth eruption in toddlers; therefore, there is still insufficient scientific research on the dynamic changes in oral microbial diversity between deciduous tooth eruption and ECC appearance.

Based on the “ecological plaque hypothesis,” this study aimed to evaluate oral microbial diversity among toddlers aged 12–24 months and to describe the dynamic processes of colonization, development, and stabilization of the oral microflora during tooth eruption using high-throughput sequencing technology.

## Materials and Methods

### Study Participants

The study participants were 12-month-old toddlers. Between October 2012 and March 2013, our research group enrolled gestational women from three maternal and child care service centers at county level in Shanghai to investigate oral health-related quality of life during pregnancy (Lu et al., 2015). The children that the women gave birth to were subjected to follow-up starting from 12 to 24 months of age. Based on our inclusion and exclusion criteria, a total of 20 toddlers aged 12 months were included in this study. Specific inclusion criteria were as follows: toddlers with good health without genetic diseases and deformities, toddlers with tooth eruption, toddlers who had not taken any antibiotics within 2 months, and toddlers who had not received any oral treatment after birth.

The study was conducted in accordance with the Declaration of Helsinki, and the protocol was approved by the Ethics Committee of the Ninth People's Hospital, Shanghai Jiao Tong University prior to the implementation of the study (Project identification code: 201410), and written informed consent was obtained from parents of toddlers.

### Clinical Examination

Oral clinical examination of the toddlers was performed and their dental caries and oral hygiene statuses were recorded. Dental caries was classified as dental caries with cavity and early caries. Dental caries with cavity was assessed according to World Health Organization (WHO) recommendations (World Health Organization, 2013), while clinical manifestations of early caries included surfaces with opacities and discolorations when viewed as wet.

Visible plaque index (VPI) was used to evaluate oral hygiene status. VPI was scored as 0 or 1 corresponding to the absence or the presence of bacterial plaque, respectively, on two surfaces per tooth; the percentage of the surfaces with plaque was also calculated, ranging between 0 and 100%.

We next collected dental plaque biofilms for extraction of genomic DNA. Before sampling, the toddlers were subjected to fasting for 2 h. Supragingival plaques were sampled from all teeth presented in the oral cavity at ages 12, 18, and 24 months. Therefore, the same teeth were sampled from each of the participants at three follow-ups depending on whether the tooth is erupted or not. A sterile curettage was used to collect the plaque sample. Pooled plaque samples were placed immediately in a sterile Eppendorf tube containing 1 ml of sodium thiosulfate solution. The tubes were transferred in liquid nitrogen to a laboratory for preservation at −80°C.

Total genomic DNA of the ensemble samples of dental plaque biofilm was extracted using the QIAGEN QIAamp DNA mini kit (Qiagen, Hilden, Germany) according to the manufacturer's instructions. A Nanodrop 2000 ultra-micro spectrophotometer (NanoDrop Technologies, Wilmington, DE, USA) was used to assess DNA concentration and purity.

### PCR Amplification

PCR amplification of variable (V) regions (V4-V5) of 16S rDNA was performed using universal primers (515F 5′- GTGCCAGCMGCCGCGG-3′, 907R, 5′-CCGTCAATTCMTTTRAGTTT-3′). The PCR reaction parameters were as follows: pre-denaturation at 98°C for 5 min; 25 cycles of denaturation for 30 s at 98°C, annealing for 30 s at 50°C, and extension for 30 s at 72°C; a final extension for 5 min at 72°C. The PCR product was detected by 2% agarose gel electrophoresis.

### Bioinformatic Analysis

After the samples were successfully amplified, they were subjected to 16S rDNA sequencing, with a sequencing depth of at least 30,000 sequences per sample, on the Illumina Miseq PE300 sequencing platform (Illumina, San Diego, CA, USA). Valid sequences were primarily obtained via collation and filtering of the original sequence data.

With the QIIME software package, high-quality sequences were clustered into operational taxonomic units (OTUs) at a 3% dissimilarity level, and the longest sequence in each OTU was selected as the representative sequence. The taxonomic information of each OTU representative sequence was obtained from sequence databases using the BLAST method (Mount, 2007).

Rarefaction curves and sequencing depth index were used to assess the depth of sequencing, species accumulation curves were used to assess whether the sample size was sufficient, and abundance distribution curves were used to determine the microbial diversity of samples. The ACE index, Chao1 index, and Simpson index were used to evaluate microbial Alpha diversity in the samples.

Community composition analysis of the samples was performed using the QIMME software package to statistically compare OTU results with databases of community structure at the phylum and genus levels for three time points. Analysis of variance (ANOVA) with multiple comparisons was performed to compare the dominant bacterial genera of the three groups of samples and thereby assess differences in species abundance. Beta diversity was evaluated by principal components analysis (PCA). In this study, PCA was performed for taxonomic classification and species abundance at the genus level, and the PCA plots were generated using the R vegan package. Moreover, the abundance information of the 50 genera with the highest abundance was subjected to inter-species correlation analysis. This information was also subjected to cluster analysis using R software, with the results depicted in a heatmap as shown in **Figure 6**.

## Results

In this study, 20 toddlers aged 12 months were enrolled. One was lost to follow-up at 18 months and one at 24 months, leaving a total of 18 toddlers who completed all three follow-ups. At 12 months of age, all toddlers showed eruption of only deciduous anterior teeth and no deciduous molar teeth. The mean number of erupted teeth was 5.6 with a mean VPI of 0.11 ± 0.18. At 18 months of age, one toddler developed a white spot lesion on two teeth. The toddlers had a mean of 11.2 erupted teeth with a mean VPI of 0.19 ± 0.15. By 24 months of age, two toddlers had developed a white spot lesion on three teeth, and they had a mean of 15.6 erupted teeth with a mean VPI of 0.18 ± 0.12. Over the course of three follow-ups, a total of 54 samples of supragingival plaque biofilm were obtained.

### Sequence Characteristics

Sequence data collation yielded 30,221,512 effective sequences from the 54 DNA samples of supragingival plaque biofilms. After screening and optimization, 17,330,301 high-quality sequences were obtained, with a minimum of 31,276 high-quality sequences in one sample and a mean of 45,982 sequences in each sample, and were used for subsequent analysis.

### Distribution of OTUs

**Figure 1:**
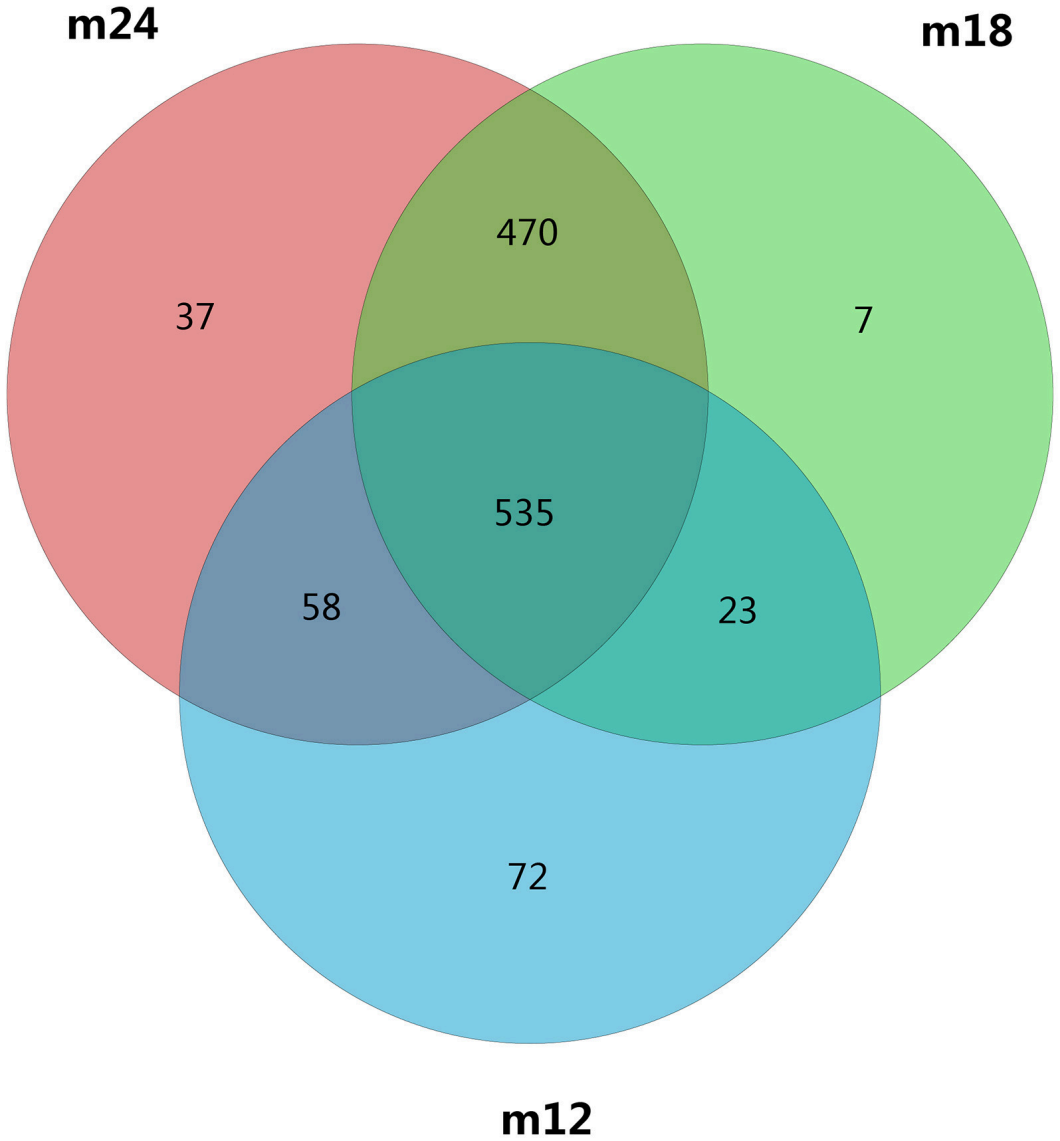
A Venn diagram showing shared and unique OTUs at 97% identity among the four groups.

OTU clustering and sequence annotation were performed using the above-obtained sequences at a 3% dissimilarity level (cutoff), and the resulting OTU tables were used for subsequent bioinformatic analysis. The OTU distributions for toddlers at 12 months of age (M12), 18 months of age (M18), and 24 months of age (M24) are shown in Figure 1. There were a total of 1202 OTUs for the three time points, with 558 in common between M12 and M18, 1005 in common between M18 and M24, 593 in common between M12 and M24, and 535 in common between M12, M18, and M24.

### Results of Species Abundance Analysis

Rarefaction curves (Figure S1A) showed that the sequencing depth was reasonable for all samples in this study, which confirmed that sequencing results reflect microbial information of the samples reasonably well. The species accumulation curves (Figure S1B) showed that the sample size in this study was reasonable. The abundance distribution curves for the 54 samples (Figure S1C) indicated that in each sample, the composition of oral microbial diversity was primarily accounted for by only a few microorganisms, while most of the other microorganisms were present in low proportions.

The Alpha diversity indices of the samples collected at the three time points are shown in Table 1. Alpha diversity indices for toddlers aged 18 and 24 months were significantly higher than those for 12-month-old toddlers (*p* < 0.001), but there was no statistical difference (*p* > 0.05) between the former two groups.

**Table 1:** Comparison of Alpha diversity among samples obtained from toddlers in 12, 18, and 24 months age groups.

	**Chao1**	**ACE**	**Simpsoneven**	**Shannon**
m12 vs. m18	*p* < 0.001	*p* < 0.001	*p* < 0.001	*p* < 0.001
m12 vs. m24	*p* < 0.001	*p* < 0.001	*p* < 0.001	*p* = 0.005
m18 vs. m24	*p* = 0.834	*p* = 1.000	*p* = 1.000	*p* = 0.876

### Results of Microbial Community Structure Analysis

**Figure 2:**
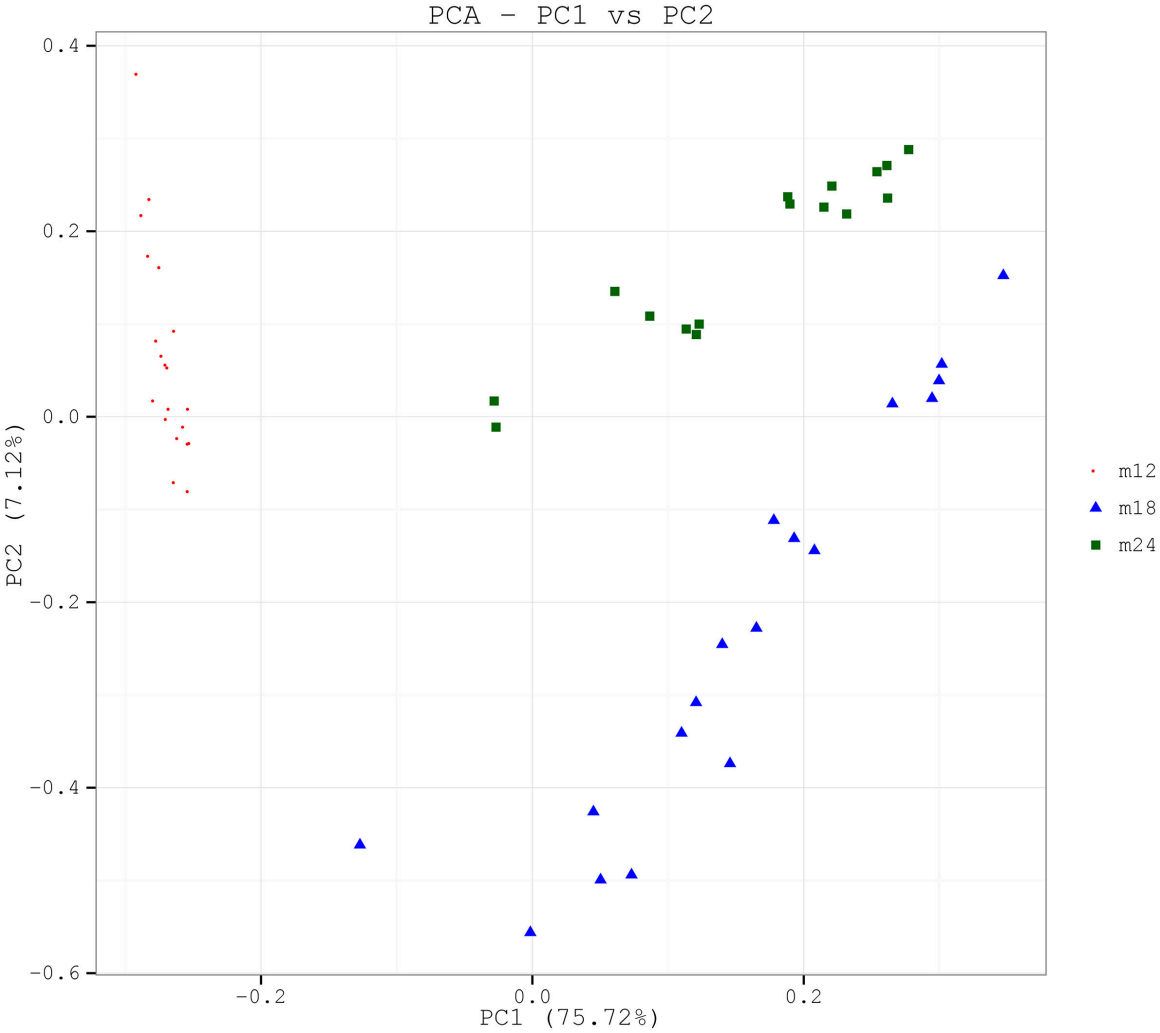
PCA based on the weighted UniFrac distances at the OUT level at 97% identity. Each sample is represented by a dot. PC1 explained 75.72% of the variation observed, and PC2 explained 7.12% of the variation. The samples formed well-separated clusters corresponding to the three groups, suggesting that the bacterial structures between groups were different.

In order to analyze the microbial community structure of the three groups of samples, PCA was performed on community composition and structure on a genus level using the software R, and the community structure results were further evaluated and compared among the three groups of samples by permutational multivariate analysis of variance (PERMANOVA) analysis. PCA analysis showed that the samples for each time point were clustered and independent without any overlapping areas (Figure 2). The distances between the samples from 18 to 24-month-old toddlers were relatively closer than between the other pairs of samples. The PERMANOVA analysis revealed that the difference between M12 and M18 (*F* = 29.029, *p* = 0.001), between M12 and M24 (*F* = 28.622, *p* = 0.001), and between M18 and M24 (*F* = 24.448, *p* = 0.001) were all statistically significant. Difference comparison based on UniFrac distances also indicated that inter-group difference in oral microbial community structure was more significant than the intra-group difference (Figure S2). In particular, the differences between the 12-month-old toddler sample and the 18-month-old toddler sample or 24-month-old toddler sample were significantly greater than the difference between the latter two.

### Results of Microbial Composition Analysis

**Figure 3:**
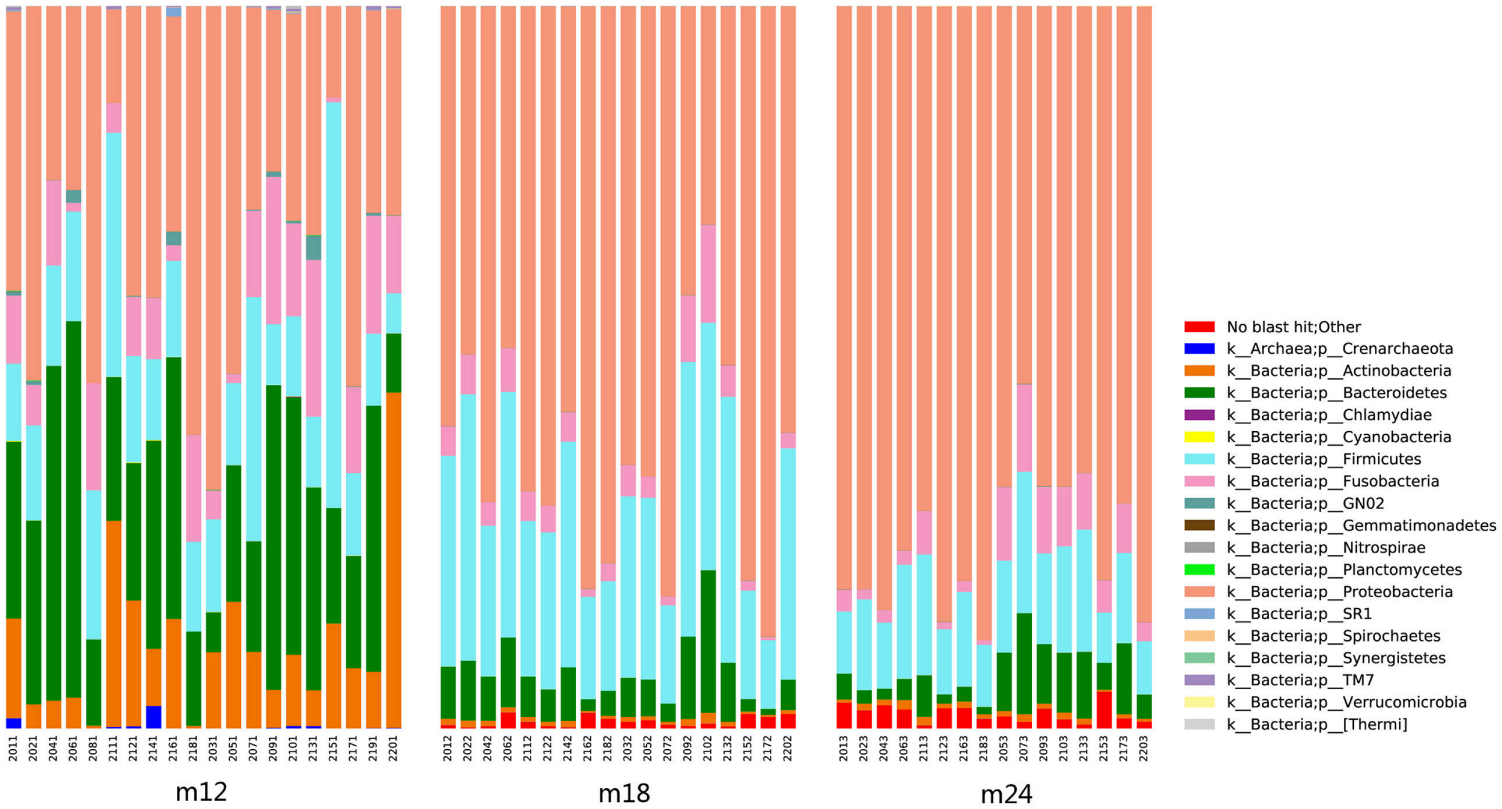
Classification and compositional ratios of the samples obtained the three time points sequenced at the taxonomic level.

Classification and compositional ratios of the samples obtained the three time points sequenced at the taxonomic level are shown in Figure 3. A total of 14 bacterial phyla were found in the samples obtained at the three time points, of which 4 were dominant: *Proteobacteria, Bacteroidetes, Firmicutes*, and *Actinobacteria* accounted for more than 80% in each sample. Comparisons of species quantity and distribution ratio among M12, M18, and M24 indicated that 11 groups of microorganisms differed at a phylum level between M12 and M18, and M12 and M24, while only 8 differed between M18 and M24. In addition, the distribution of *Proteobacteria* and *Firmicutes* in the toddlers was significantly lower in M12 than in M18 and M24, whereas the distribution of *Bacteroidetes* and *Actinobacteria* was significantly higher in the former group than in the latter two. By contrast, the above 4 groups of microorganisms did not show significant differences between M18 and M24 in terms of their distribution.

**Figure 4:**
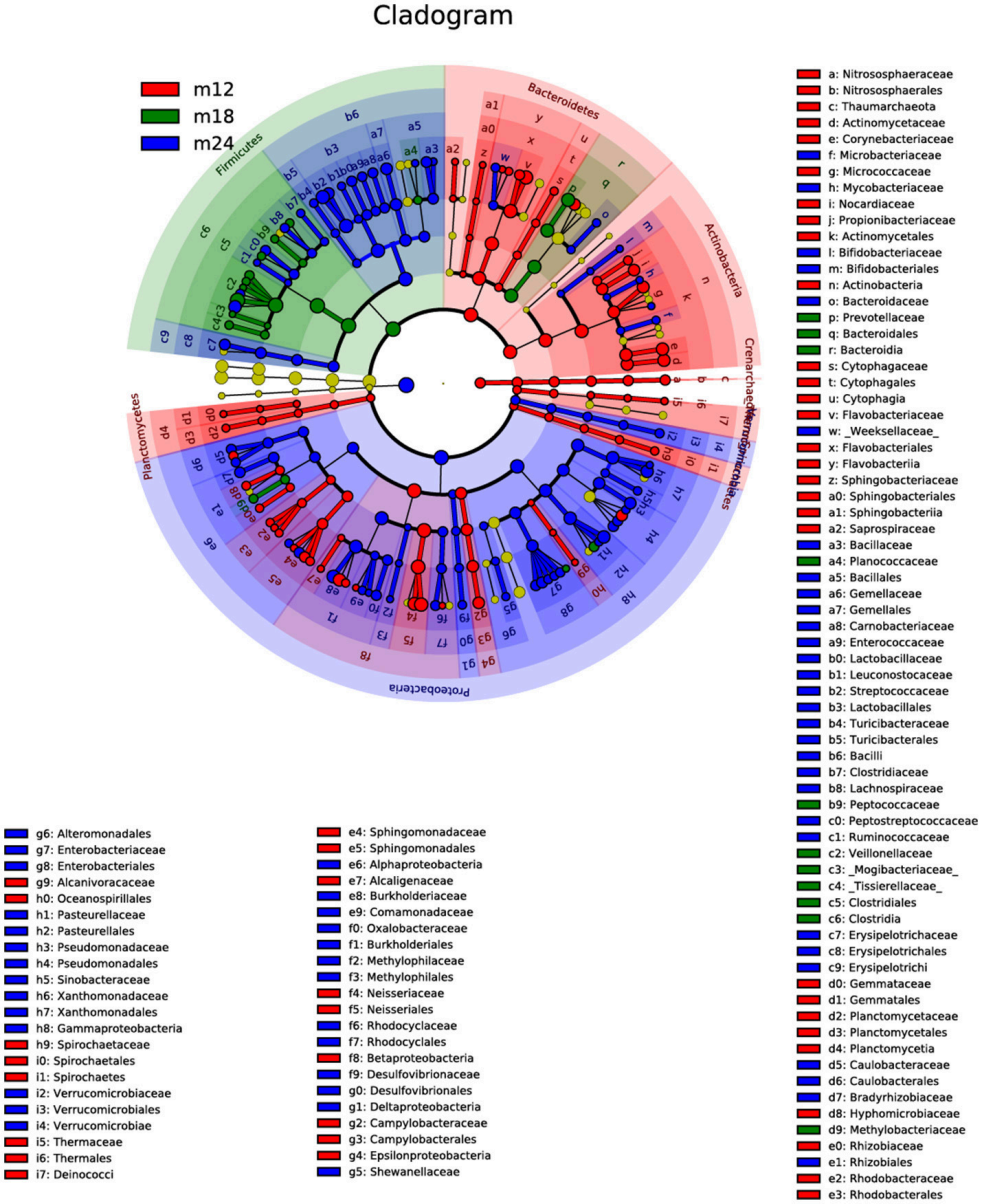
The linear discriminant analysis effect size (LEfSe). The colored nodes from the inner to outer circles represent the top abundant taxa from phylum to genus level. The yellow nodes represent taxa showing no significant difference between groups, while nodes with other colors represent taxa showing significant difference between groups.

These results agree with the results of the linear discriminant analysis effect size (LEfSe) (Figure 4). Comparison of the abundance of dominant genera among samples from the three time points showed that the dominant genera in M12 were *Bacteroidetes* and *Actinobacteria* but shifted to *Firmicutes* in M18 and to *Proteobacteria* in M24. The M18 group had *Bacteroidetes* in common with the M12 group but *Firmicutes* in common with the M24 group. The species that were present in all the three groups were *Bacteroidia* of phylum *Bacteroidetes* and *Rhizobiales* of phylum *Proteobacteria*.

Dominant genera abundances were subjected to difference analysis using the compositional ratios at a phylum level in the three samples(Figure S3). A total of 192 different genera were identified in all the samples, including 20 phyla of bacteria accounting for more than 80% of the obtained sequences, namely *Unclassified_Enterobacteriaceae, Selenomonas, Neisseria, Capnocytophaga, Streptococcus, Lautropia, Leptotrichia, Prevotella, Veillonella, Burkholderia, Fusobacterium, Stenotrophomonas, Actinomyces, Kingella, Corynebacterium, Haemophilus, Tannerella, Rothia, Porphyromonas, Gemella*, and *Campylobacter*. At a genus level, there were 82 and 79 microbial species that differed between the M12 and M18 samples and between the M12 and M24 samples, respectively, whereas 96 species differed between the M18 and M24 samples.

*Neisseria* (with an overall percentage of 13.4%), *Streptococcus* (12.0%), *Leptotrichia* (7.9%), *Prevotella* (5.4%), and *Kingella* (5.0%) were all present at relatively stable and high percentages in all 54 samples, thereby representing the core genera for all three samples. However, *Capnocytophaga, Lautropia, Corynebacterium, Actinomyces*, and *Rothia*, which were present in relatively high percentages in the early period, continued to decrease in the M18 and M24 groups of samples.

*Unclassified_Enterobacteriaceae, Burkholderia*, and *Stenotrophomonas* were present in extremely low proportions in M12 samples, but their percentages gradually increased in M18 and M24 samples. These trends are likely to represent the order of bacterial colonization in the oral cavity of toddlers during the process of plaque maturation.

### RDA Analysis

**Figure 5:**
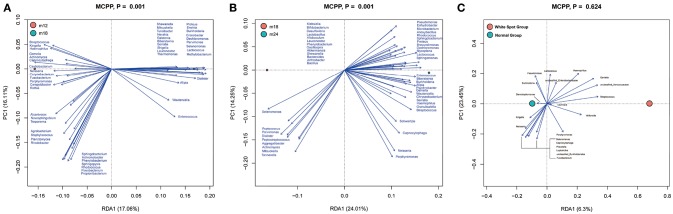
Redundancy analysis (RDA). Bioplot of RDA of the microbiota between 12 and 18 m **(A)**, 18 and 24 m **(B)** samples, responding the OTUs with at least 13 **(A)** and 15% **(B)** of the variability of the oral microbiota are indicated by blue arrows. Bioplot of RDA of the microbiota relative to the early dental caries **(C)**, responding the OTUs with at least 16% of the variability of the oral microbiota.

To analyze key phylotypes affecting early dental caries, the software R was used to perform a two-step redundancy analysis (RDA) on the relative abundance matrix at the genus level (Figures 5A,B). The first step was to use toddler samples from the three follow up time points as an environmental variable and the relative abundance of OTUs within the samples as a species variable. The Monte Carlo Permutation Procedure (MCPP) showed that there was a statistically significant difference (*p* = 0.001) in species between M12 and M18 as well as between M18 and M24. There were 50 key phylotypes accounting for 13% of the species difference between M12 and M18, and 15% between M18 and M24.

Furthermore, in order to determine whether the presence of early dental caries (white spot lesion) was an influential factor, the M24 group of samples was divided into two sub-groups based on the presence or absence of early dental caries and subjected to RDA analysis (Figure 5C). The MCPP results showed that the two sub-groups did not statistically differ in terms of species (*p* = 0.624).

**Figure 6:**
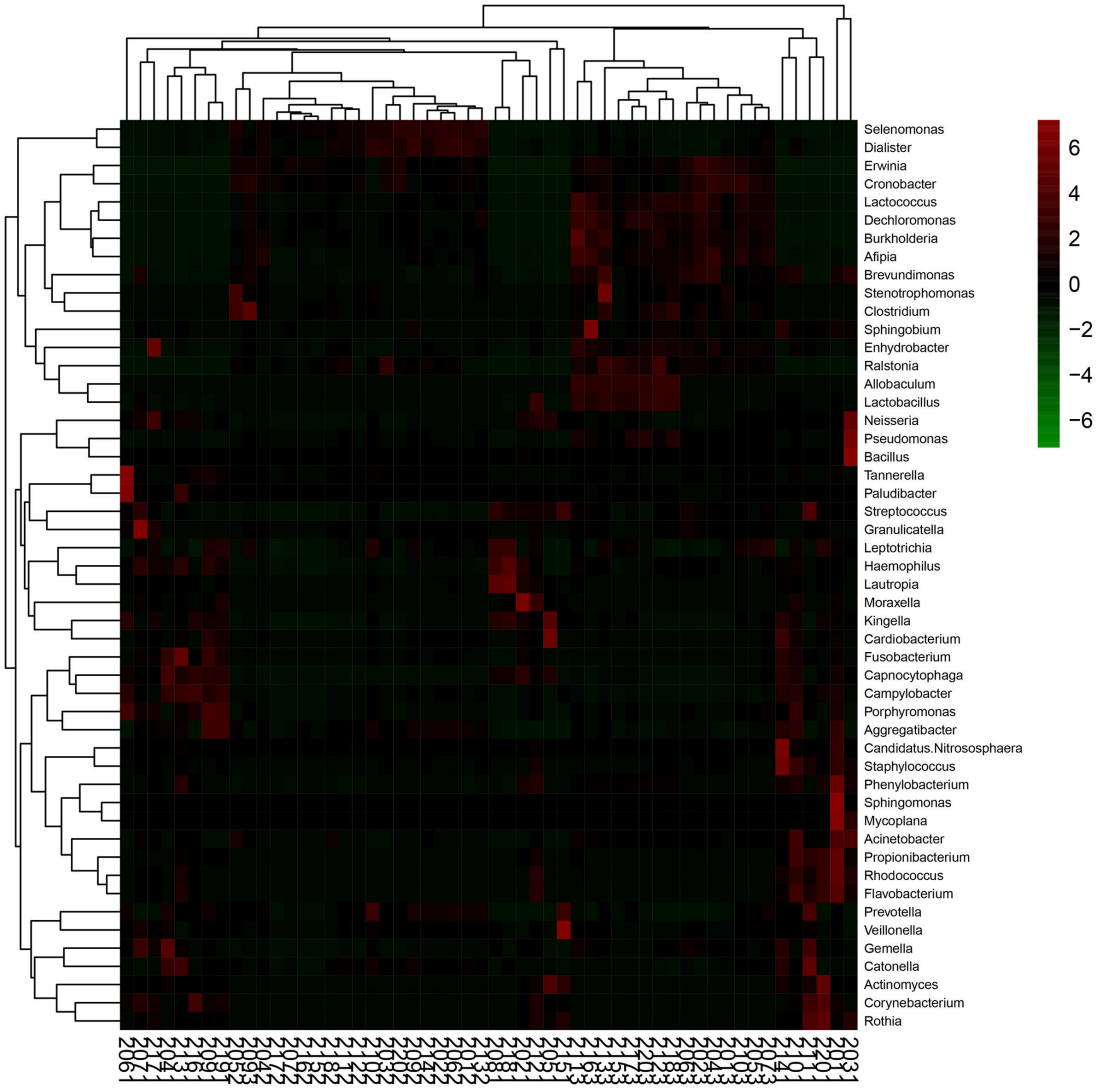
Heat-map of the 50 relative abundant key bacteria genera. The color of the spot corresponds to the normalized relative abundances of the genera.

The top 50 genera of bacteria arranged in descending order of abundance that were used in RDA to assess the differences among the three groups of samples were subjected to heatmap analysis as shown in Figure 6. The results indicated that the M12 samples were relatively dispersive, primarily containing *Capnocytophaga, Streptococcus, Neisseria, Campylobacter, Prevotella*, and *Veillonella*, with high relative abundance and cluster richness. By the age of 18 and 24 months, the clustering level was relatively high, and the high-abundance species areas of the two groups were closely situated, concentrated around *Selenomonas, Lactococcus, Erwinia*, and *Cronobacter*. This showed that the types of species that colonize the oral cavity of toddlers are complex and that they exhibit variations and instability between individuals. After the eruption of dentition and the gradual establishment of occlusion, oral microflora composition becomes relatively stable.

## Discussion

Oral microflora is a major component of the oral ecosystem. The establishment and succession of normal microflora are closely related to the age of the host, feeding habits, oral hygiene habits, and the parents' indigenous oral microflora (Filoche et al., 2010). During the final process of deciduous dentition in toddlers, the microflora in the oral cavity also undergo changes based on the microecological balance in the oral cavity. The period between 12 and 24 months of age is crucial for the establishment of oral cavity microflora owing to changes induced by the processes of tooth eruption and dentition. Investigation of the characteristics of early colonizing bacteria in the oral cavity of toddlers will help understand the characteristics and balance of microflora balance in the oral cavity so as to prevent dysbiosis-induced infectious diseases such as dental caries, which is directly caused by opportunistic pathogens.

Given technological limitations, past research on oral microorganisms has involved bacterial culture to investigate the association of a single or several bacteria with oral diseases such as ECC (Aas et al., 2005). In recent years, increasing evidence indicates that microorganisms in the environment function as an organic whole to affect the host's health status, with local micro-ecological disorders and the destruction of ecological balance among bacteria often leading to the occurrence of disease (Hajishengallis and Lamont, 2012; Human Microbiome Project C, 2012). To date, 687 oral microorganisms have been reported in the Human Oral Microbiome Database (HOMD) (Chen et al., 2010), and studies have shown that only 50% of the total bacteria from the oral cavity can be cultivated under experimental conditions (Aas et al., 2005). Research on such a complex ecosystem requires more advanced technology. The advent of non-culture-dependent metagenomic technologies has made it possible to rapidly detect changes in microbial communities (Chen et al., 2015; Xiao et al., 2016). In this study, high-throughput sequencing and biological informatics were used for cohort analysis of samples from toddlers aged 12–24 months, which provided an in-depth insight into the oral bacterial development of toddlers.

The oral cavity of a baby undergoes several environmental changes. With the formation of the periodontal pocket during primary tooth eruption, an anaerobic niche develops in the oral cavity, which make it possible for the colonization of anaerobes (Caufield et al., 2000). These processes stimulate corresponding changes in the nature and composition of oral microflora, which gradually stabilize with the eruption of teeth. Previous studies on the oral microflora of babies mostly focused on the development of oral microflora during the neonatal period (0–1 years old) (Rosenblatt et al., 2015; Chu et al., 2017) and preschool age (3–6 years old) (Ling et al., 2010; Zhang et al., 2015) with very little focus on the development of oral microflora in children during the teething period. The results of this study confirmed from several experimental angles that the process of dentition in toddlers is accompanied by high dynamism in oral microbial communities.

The Alpha diversity index showed that toddlers have greater microflora species diversity at 18 and 24 months of age than at 12 months of age. PCA showed significant differences in constituent microbial species among samples taken from toddlers belonging to the three different age groups. LEfSe analysis results indicated that the core species of the samples were both similar and different among the three age groups. With increasing age, the 20 dominant genera accounting for 80% of the total number of species continued to change in abundance ratios. In 12-month-olds, *Capnocytophaga, Neisseria, Streptococcus, Kingella*, and *Leptotrichia* were the main species; at 18 and 24 months of age, the abundance ratio of *Capnocytophaga* continued to decrease but those of *Burkholderia* and *Stenotrophomonas* showed an increasing trend, and *Enterobacteriaceae*, in particular, became a dominant genus. Despite these differences, *Streptococcus, Neisseria*, and *Leptotrichia* were consistently found to be the dominant genera, with a high level of abundance in toddlers from 12 to 24 months of age. The above results indicate that microflora colonization in the oral cavity of toddlers during the teething period is a dynamic and continuous process involving certain core species that remain dominant, with different species gathering and colonizing around the core species at different ages to eventually reach microbial balance through a series of complex processes including symbiosis, competition, and inhibition.

The results of this study showed that during growth from 18 and 24 months, the community structure and composition of the oral bacteria remained similar. There were 1005 OTUs in common between the 18 and 24-months age groups, which was much higher than the number of OTUs in common between 18 and 12-months age groups. The Alpha diversity index showed that compared with the difference in microbial diversity between 12 and 18 months of age, no significant difference was observed between 18 and 24 months of age. Of the seven core genera with the highest abundance in toddlers aged 18 months, six were also included among the seven most abundant genera in toddlers aged 24 months; in addition, both PCA and UniFrac analysis indicated a relatively tight connection between microbial communities in 18 and 24-month-olds. This shows that compared with younger babies, the oral bacterial community structure in toddlers aged 18 to 24 months began to stabilize after the eruption of most of their teeth, during the gradual establishment of occlusion. These results were consistent with the findings of Sarkonen (Sarkonen et al., 2000).

As early as 1965, McCarthy(McCarthy et al., 1965) found that *Streptococcus, Staphylococcus*, and *Neisseria* were the dominant bacteria in the oral cavity of 12-month-old infants. With the eruption of teeth, the rate at which *Actinomycetes* and *Fusobacteria* were detected increased. Kononen also found in 1999 that *Capnocytophaga* was an early dominant genus in the oral cavity of infants (Könönen et al., 1999). A study by Zou et al. in 2004 found that *Streptococcus* was a dominant genus in the oral cavity of infants <1-year-old (Zou et al., 2004), and the mouth also contained anaerobes such as *Capnocytophaga*. These findings were consistent with the results of this study—that the core genera previously described to colonize the oral cavity early, namely *Streptococcus, Neisseria*, and *Capnocytophaga*, were found in the samples of 12-month-old toddlers analyzed in our study. From 18 to 24 months, with the increase of teeth erupted, proportion of Gram-negative bacillus declined (Selenomonas from 20.6 to 2.9% and Prevotella from 5.1 to 2.1%), while the Gram-negative cocci rose (*Streptococcus* and *Burkholderiales*). This variation was also consistent with the former reports (Zou et al., 2004; Shi et al., 2016). The microbial composition and structure of 24 months toddlers were more similar to that of deciduous dentition children aged 3–5 years (Xu et al., 2015).

Moreover, species of *Streptococcus* and *Neisseria* appeared as core species all three age groups and accounted for a high proportion of detected species, which was consistent with previous findings (Tao et al., 2013) and once again proved that *Streptococcus* and *Neisseria* are early and major members of the oral microflora in toddlers. *Actinomyces* are autochthonous or early colonizing microorganisms in the oral cavity, and are associated with the occurrence of dental caries in young children and involved in the formation of early plaques (Sarkonen et al., 2000). This is consistent with the results of our study, where we found a high abundance of *Actinomyces* in the oral plaque samples collected from 12-month-old toddlers.

In this study, the unclassified Enterobacteriaceae genus in the family of *Enterobacteriaceae* accounted for more than 50% of the total number of genera in the samples from toddlers aged 18 and 24 months, making it a dominant genus in the oral cavity. We found no related reports in the literature. This may be because the annotation in this study failed to accurately classify bacteria belonging to the *Enterobacteriaceae* family, causing the category unclassified Enterobacteriaceae to contain information from multiple genera. It may also be attributed to the fact that during the process of maturation, the intestinal microflora of toddlers is significantly associated with oral microflora. Further research is needed to explore why unclassified Enterobacteriaceae of *Enterobacteriaceae* is dominant in the oral microflora of toddlers.

White spot lesion was observed in toddlers aged 24 months, but no cavity was found. Therefore, we divided the samples into two sub-groups based the presence of white spot lesions and tried to analyze whether specific dominant bacteria were differentially present in the early stages of ECC. However, RDA failed to detect significant species differences between toddlers with white spot lesion and toddlers without, which may be due to the fact that assemble samples were collected from the mouth, resulting in insufficient site-specificity, which in turn led to insignificant species differences between the two sub-groups. Reports have shown that the onset of ECC in Chinese infants was detected in 20-month old infants (Zhou et al., 2012), which appears to be later than that in other countries (Thitasomakul et al., 2006). In future research, more site-specific specimens should be collected.

Possible limitation of the present study should be considered. First of all, the observational period is short. At 24 months of age, all the toddlers had not yet developed dental caries, which made it difficult to observe the endpoint of microflora succession and analyze early cariogenic bacteria. Secondly, some external factors, such as the feeding habits, oral hygiene habits, and the parents' indigenous oral microflora, may associate with the progression of the oral microbiota of toddlers from 12 to 24 months. However, due to the lack of this information in the present study, the role of external factors may not be investigated. In the future, it is necessary to extend the observational period, improve sampling methods and collect more comprehensive information to describe the overall profile of oral microbiota among toddlers.

## Author Contributions

HL, MW, and XF conceived and designed the experiments. FL, DT, and HL performed the experiments. HL, MW, and XF analyzed the data. FL, DT, MW, and HL contributed reagents, materials, and analysis tools. FL, DT, and HL wrote the paper. XF and MW assisted with the full text and revised the grammar.

### Conflict of Interest Statement

The authors declare that the research was conducted in the absence of any commercial or financial relationships that could be construed as a potential conflict of interest.

## Supplementary Material

Supplementary Figure 1 Quality control of the sequencing. **(A)** The rarefaction curves. **(B)** The species accumulation curves. **(C)** The abundance distribution curves.

Supplementary Figure 2 UniFrac The cross ordinates correspond to the statistical comparisons between groups, and the longitudinal coordinates indicate the corresponding distance values.

Supplementary Figure 3 Classification and compositional ratios of the samples obtained the three time points sequenced at the taxonomic level.

## References

[B1] Aas J. A. Paster B. J. Stokes L. N. Olsen I. Dewhirst F. E. (2005). Defining the normal bacterial flora of the oral cavity. J. Clin. Microbiol. 43, 5721–5732. 10.1128/JCM.43.11.5721-5732.2005 16272510 PMC1287824

[B2] Carlsson J. Grahnen H. Jonsson G. Wikner S. (1970). Establishment of Streptococcus sanguis in the mouths of infants. Arch. Oral Biol. 15, 1143–1148. 10.1016/0003-9969(70)90005-1 5280120

[B3] Caufield P. W. Dasanayake A. P. Li Y. Pan Y. Hsu J. Hardin J. M. (2000). Natural history of Streptococcus sanguinis in the oral cavity of infants: evidence for a discrete window of infectivity. Infect Immun. 68, 4018–4023. 10.1128/IAI.68.7.4018-4023.2000 10858217 PMC101685

[B4] Chen H. Liu Y. Zhang M. Wang G. Qi Z. Bridgewater L.. (2015). A Filifactor alocis-centered co-occurrence group associates with periodontitis across different oral habitats. Sci Rep. 5:9053. 10.1038/srep09053 25761675 PMC4356962

[B5] Chen T. Yu W. H. Izard J. Baranova O. V. Lakshmanan A. Dewhirst F. E. (2010). The Human Oral Microbiome Database: a web accessible resource for investigating oral microbe taxonomic and genomic information. Database 2010:baq013. 10.1093/database/baq013 20624719 PMC2911848

[B6] Chu D. M. Ma J. Prince A. L. Antony K. M. Seferovic M. D. Aagaard K. M. (2017). Maturation of the infant microbiome community structure and function across multiple body sites and in relation to mode of delivery. Nat. Med. 23, 314–326. 10.1038/nm.4272 28112736 PMC5345907

[B7] Filoche S. Wong L. Sissons C. H. (2010). Oral biofilms: emerging concepts in microbial ecology. J. Dent. Res. 89, 8–18. 10.1177/0022034509351812 19918089

[B8] Hajishengallis G. Lamont R. J. (2012). Beyond the red complex and into more complexity: the polymicrobial synergy and dysbiosis (PSD) model of periodontal disease etiology. Mol Oral Microbiol. 27, 409–419. 10.1111/j.2041-1014.2012.00663.x 23134607 PMC3653317

[B9] Human Microbiome Project C (2012). Structure, function and diversity of the healthy human microbiome. Nature 486, 207–214. 10.1038/nature11234 22699609 PMC3564958

[B10] Könönen E. Kanervo A. Takala A. Asikainen S. Jousimies-Somer H. (1999). Establishment of oral anaerobes during the first year of life. J Dent Res. 78, 1634–1639. 10.1177/00220345990780100801 10520968

[B11] Ling Z. Kong J. Jia P. Wei C. Wang Y. Pan Z.. (2010). Analysis of oral microbiota in children with dental caries by PCR-DGGE and barcoded pyrosequencing. Microb. Ecol. 60, 677–690. 10.1007/s00248-010-9712-8 20614117

[B12] Lu H. X. Xu W. Wong M. C. Wei T. Y. Feng X. P. (2015). Impact of periodontal conditions on the quality of life of pregnant women: a cross-sectional study. Health Qual. Life Outcomes 13:67. 10.1186/s12955-015-0267-8 26018650 PMC4446953

[B13] Marsh P. D. (1994). Microbial ecology of dental plaque and its significance in health and disease. Adv. Dent. Res. 8, 263–271. 10.1177/08959374940080022001 7865085

[B14] McCarthy C. Snyder M. L. Parker R. B. (1965). The indigenous oral flora of man. i. the newborn to the 1-year-old infant. Arch. Oral Biol. 10, 61–70. 14262161 10.1016/0003-9969(65)90058-0

[B15] Mount D. W. (2007). Using the Basic Local Alignment Search Tool (BLAST). CSH Protoc. 1 2007:pdb top17. 21357135 10.1101/pdb.top17

[B16] Rosenblatt R. Steinberg D. Mankuta D. Zini A. (2015). Acquired oral microflora of newborns during the first 48 hours of life. J. Clin. Pediatr. Dent. 39, 442–446. 10.17796/1053-4628-39.5.442 26551367

[B17] Sarkonen N. Kononen E. Summanen P. Kanervo A. Takala A. Jousimies-Somer H. (2000). Oral colonization with Actinomyces species in infants by two years of age. J Dent Res. 79, 864–867. 10.1177/00220345000790031301 10765961

[B18] Shi W. Qin M. Chen F. Xia B. (2016). Supragingival microbial profiles of permanent and deciduous teeth in children with mixed dentition. PLoS ONE 11:e0146938. 10.1371/journal.pone.0146938 26752284 PMC4709228

[B19] Tao Y. Zhou Y. Ouyang Y. Lin H. (2013). Dynamics of oral microbial community profiling during severe early childhood caries development monitored by PCR-DGGE. Arch Oral Biol. 58, 1129–1138. 10.1016/j.archoralbio.2013.04.005 23664249

[B20] Thitasomakul S. Thearmontree A. Piwat S. Chankanka O. Pithpornchaiyakul W. Teanpaisan R.. (2006). A longitudinal study of early childhood caries in 9- to 18-month-old Thai infants. Commun. Dent Oral Epidemiol. 34, 429–436. 10.1111/j.1600-0528.2006.00292.x 17092271

[B21] Whiley R. A. Beighton D. (1998). Current classification of the oral streptococci. Oral Microbiol. Immunol. 13, 195–216. 10.1111/j.1399-302X.1998.tb00698.x 10093535

[B22] World Health Organization (2013). Oral Health Surveys: Basic Methods. 5th ed Geneva: WHO.

[B23] Xiao C. Ran S. Huang Z. Liang J. (2016). Bacterial diversity and community structure of supragingival plaques in adults with dental health or caries revealed by 16S pyrosequencing. Front Microbiol. 7:1145. 10.3389/fmicb.2016.01145 27499752 PMC4956651

[B24] Xu X. He J. Xue J. Wang Y. Li K. Zhang K.. (2015). Oral cavity contains distinct niches with dynamic microbial communities. Environ. Microbiol. 17, 699–710. 10.1111/1462-2920.12502 24800728

[B25] Zhang M. Chen Y. Xie L. Li Y. Jiang H. Du M. (2015). Pyrosequencing of plaque microflora in twin children with discordant caries phenotypes. PLoS ONE 10:e0141310. 10.1371/journal.pone.0141310 26524687 PMC4629883

[B26] Zhou Y. Yang J. Y. Lo E. C. Lin H. C. (2012). The contribution of life course determinants to early childhood caries: a 2-year cohort study. Caries Res. 46, 87–94. 10.1159/000335574 22343693

[B27] Zou J. Zhou X. D. Li S. M. (2004). Analysis of oral microflora early colonized in infants. Hua Xi Kou Qiang Yi Xue Za Zhi. 22, 126–128. 10.3321/j.issn:1000-1182.2004.02.013 15190795

