# Supplementary figures and images for: Establishment and Development of Oral Microflora in 12–24 Month-Old Toddlers Monitored by High-Throughput Sequencing
Source: Front Cell Infect Microbiol. 2018 Dec 4;8:422. doi: 10.3389/fcimb.2018.00422 (PMC6288402; doi:10.3389/fcimb.2018.00422)

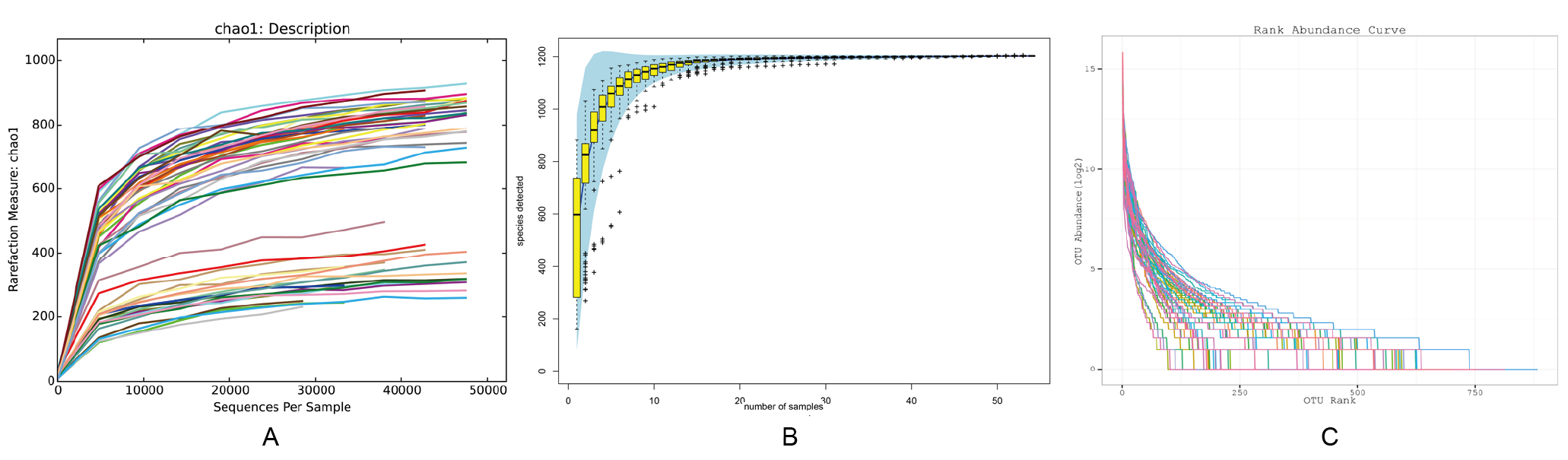

Supplement: Supplementary Figure 1 — Quality control of the sequencing. (A) The rarefaction curves. (B) The species accumulation curves. (C) The abundance distribution curves. [file Image_1.TIF]

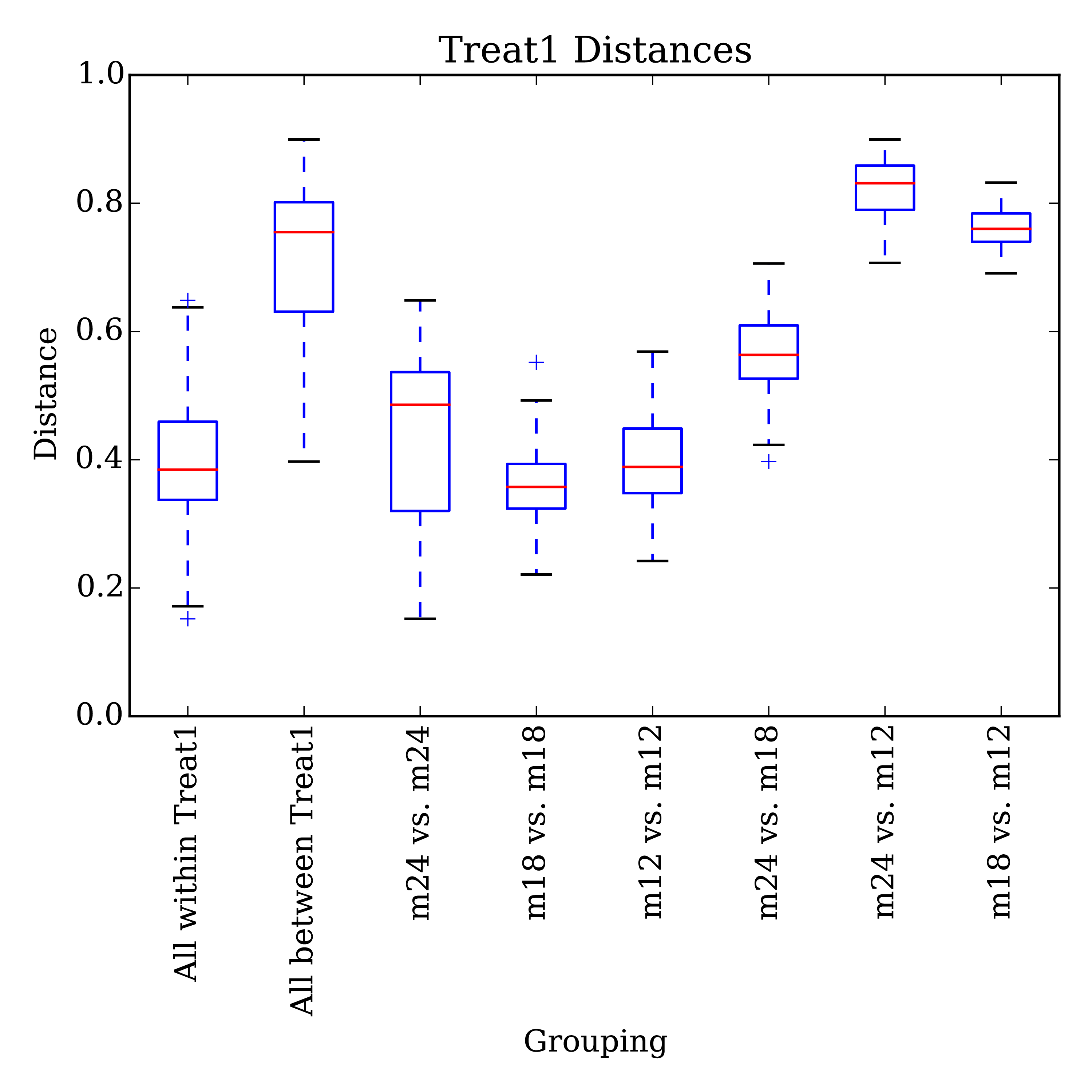

Supplement: Supplementary Figure 2 — UniFrac The cross ordinates correspond to the statistical comparisons between groups, and the longitudinal coordinates indicate the corresponding distance values. [file Image_2.TIF]

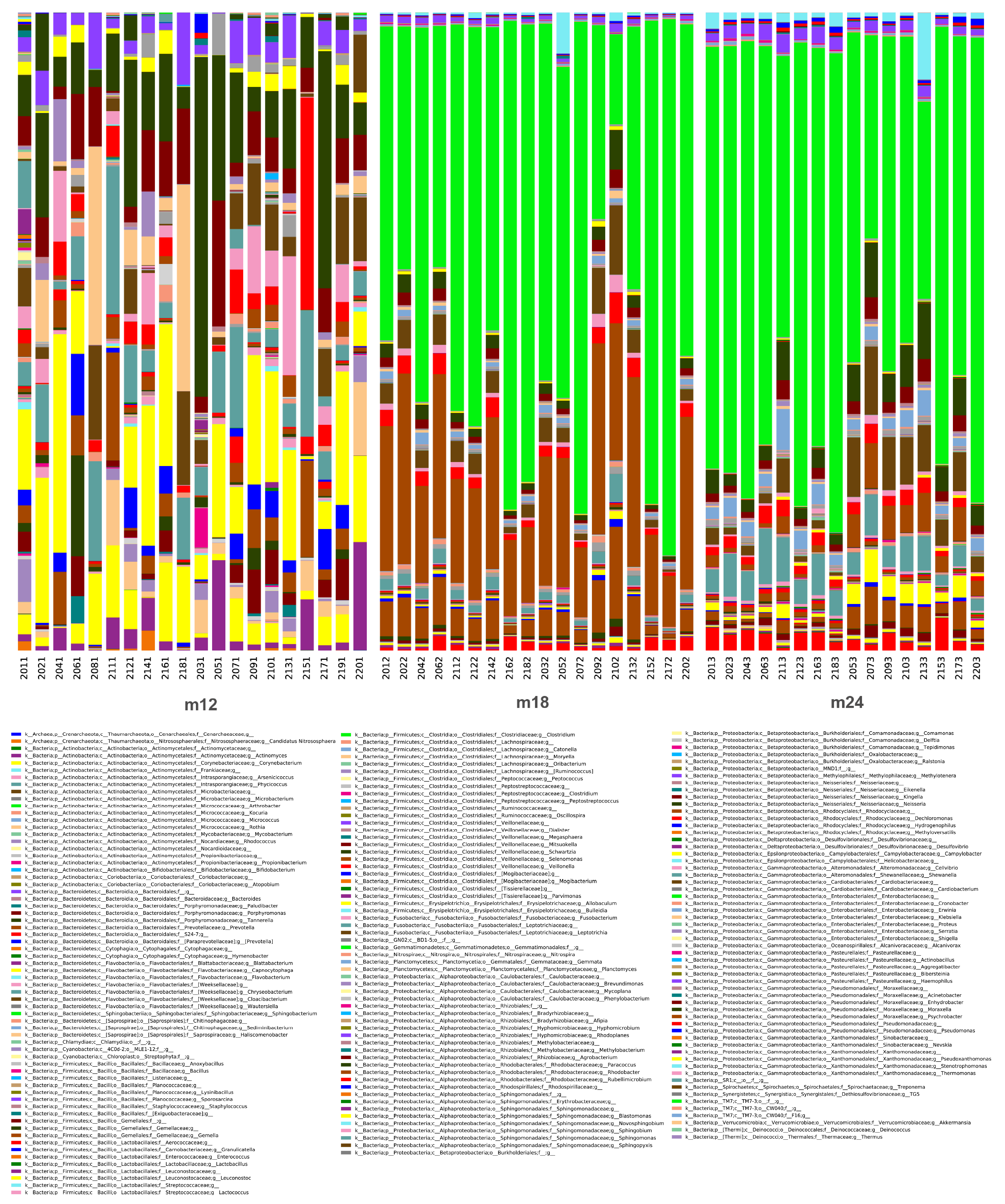

Supplement: Supplementary Figure 3 — Classification and compositional ratios of the samples obtained the three time points sequenced at the taxonomic level. [file Image_3.TIF]
